# Supplementary material for: Effects of Selective and Mixed-Action Kappa and Delta Opioid Receptor Agonists on Pain-Related Behavioral Depression in Mice
Source: Molecules. 2024 Jul 16;29(14):3331. doi: 10.3390/molecules29143331 (PMC11279860; doi:10.3390/molecules29143331)

SUPPLEMENTAL MATERIAL FOR

Effects of Selective and Mixed-Action Kappa and Delta Opioid Receptor Agonists on Pain-Related Behavioral Depression in Mice

S. Stevens Negus[1*], Celsey M. St. Onge[2], Young K. Lee[1], Mengchu Li[2], Kenner C. Rice[3] and Yan Zhang[2]

[1] Department of Pharmacology and Toxicology, School of Medicine, Virginia Commonwealth University, Richmond VA, 23298, United States

[2] Department of Medicinal Chemistry, School of Pharmacy, Virginia Commonwealth University, Richmond VA 23298, United States

[3] Drug Design and Synthesis Section, Molecular Targets and Medications Discovery Branch, NIDA and NIAAA, Bethesda, MD, United States

* Correspondence: sidney.negus@vcuhealth.org; Tel.: 804-828-3158

Supplemental Table S1. Two-way ANOVA results for the main effect of Sex and for the Dose x Sex interaction for effects of each drug on Crosses and Movement when the drug was administered alone or as a pretreatment to IP 0.56% lactic acid. *p<0.05.

Test Drug and Condition	Sex	Dose x Sex
Hydrocodone		
Alone Crosses	F(1,50)=6.34, p=0.0150*	F(4,50)=0.91, p=0.4619
Alone Movement	F(1,50)=1.89, p=0.1754	F(4,50)=1.06, p=0.3887
+ IP Acid Crosses	F(1,70)=1.04, p=0.3125	F(6,70)=0.89, p=0.5092
+ IP Acid Movement	F(1,70)=0.01, p=0.9251	F(6,70)=0.84, p=0.5447
Aprepitant		
Alone Crosses	F(1,50)=0.02, p=0.8754	F(4,50)=5.44, p=0.0010*
Alone Movement	F(1,50)=1.92, p=0.1714	F(4,50)=0.65, p=0.6318
+ IP Acid Crosses	F(1,50)=14.00, p=0.0005*	F(4,50)=2.28, p=0.0733
+ IP Acid Movement	F(1,50)=0.21, p=0.6512	F(4,50)=2.51, p=0.0535
Nalfurafine		
Alone Crosses	F(1,60)=4.46, p=0.0389*	F(5,60)=1.85, p=0.1174
Alone Movement	F(1,60)=0.89, p=0.3491	F(5,60)=1.33, p=0.2637
+ IP Acid Crosses	F(1,60)=8.90, p=0.0041*	F(5,60)=1.04, p=0.4052
+ IP Acid Movement	F(1,60)=5.04, p=0.0284*	F(5,60)=0.62, p=0.6853
SNC80		
Alone Crosses	F(1,70)=0.14, p=0.7091	F(6,70)=1.36, p=0.2408
Alone Movement	F(1,70)=5.84, p=0.0183*	F(6,70)=0.71, p=0.6419
+ IP Acid Crosses	F(1,100)=4.00, p=0.0483*	F(9,100)=2.08, p=0.0380*
+ IP Acid Movement	F(1,100)=2.02, p=0.1584	F(9,100)=2.37, p=0.0179*
10:1 SNC80/Nalfurafine		
Alone Crosses	F(1,60)=0.10, p=0.7479	F(5,60)=1.54, p=0.1898
Alone Movement	F(1,60)=0.07, p=0.7983	F(5.60)=0.76, p=0.5802
+ IP Acid Crosses	F(1,70)=0.02, p=0.8880	F(6,70)=1.60, p=0.1602
+ IP Acid Movement	F(1,70)=0.27, p=0.6074	F(6,70)=1.03, p=0.4106
TK10		
Alone Crosses	F(1,50)=1.01, p=0.3196	F(4,50)=0.36, p=0.8348
Alone Movement	F(1,50)=5.41, p=0.0242*	F(4,50)=0.53, p=0.7148
+ IP Acid Crosses	F(1,50)=0.05, p=0.8303	F(4,50)=0.18, p=0.9465
+ IP Acid Movement	F(1,50)=2.80, p=0.1003	F(4,50)=0.43, p=0.7832
TK33		
Alone Crosses	F(1,50)=0.90, p=0.3464	F(4,50)=0.87, p=0.4896
Alone Movement	F(1,50)=0.27, p=0.6045	F(4,50)=0.49, p=0.7427
+ IP Acid Crosses	F(1,50)=5.09, p=0.0285*	F(4,50)=0.52, p=0.7241
+ IP Acid Movement	F(1,50)=1.75, p=0.1923	F(4,50)=0.53, p=0.7129
TK35		
Alone Crosses	F(1,44)=0.89, p=0.3518	F(4,44)=1.72, p=0.1632
Alone Movement	F(1,44)=9.46, p=0.0036*	F(4,44)=2.00, p=0.1109
+ IP Acid Crosses	F(1,50)=2.87, p=0.0968	F(4,50)=1.46, p=0.2273
+ IP Acid Movement	F(1,50)=3.74, p=0.0590	F(4,50)=2.86, p=0.0327*

Supplemental Table S2. Post hoc power analysis of Dose x Sex interactions for each drug on Crosses and Movement when the drug was administered alone or as a pretreatment to IP 0.56% lactic acid. Eta^2 values for effect size were calculated from Prism ANOVA tables and entered into G*Power to determine Cohen's F Effect Size, Current Power (1-ß) with the existing sample size, and estimated sample size to detect a significant effect with Power ≥0.8 as described previously [49]. Note that "Current Sample Size" equals the sample size for each dose (N=12 for all conditions except one dose of TK35 alone) times the number of doses.

Test Drug and Condition	Effect Size (eta^2)	Cohen's Effect Size (Cohen's F)	Current Power	Current Sample Size	Sample Size for Power≥0.8
Hydrocodone					
Alone Crosses	0.068	0.270	0.319	60	169
Alone Movement	0.078	0.290	0.368	60	147
+ IP Acid Crosses	0.071	0.276	0.395	84	185
+ IP Acid Movement	0.067	0.268	0.371	84	197
Aprepitant					
Alone Crosses	0.303	0.659	0.985	60	34
Alone Movement	0.049	0.227	0.231	60	>200
+ IP Acid Crosses	0.154	0.427	0.713	60	71
+ IP Acid Movement	0.167	0.448	0.760	60	65
Nalfurafine					
Alone Crosses	0.133	0.392	0.681	72	90
Alone Movement	0.100	0.333	0.521	72	122
+ IP Acid Crosses	0.079	0.293	0.408	72	156
+ IP Acid Movement	0.049	0.227	0.249	72	>200
SNC80					
Alone Crosses	0.105	0.343	0.591	84	123
Alone Movement	0.057	0.246	0.313	84	>200
+ IP Acid Crosses	0.158	0.433	0.915	120	94
+ IP Acid Movement	0.176	0.462	0.950	120	84
10:1 SNC80/Nalfurafine					
Alone Crosses	0.114	0.359	0.592	72	106
Alone Movement	0.060	0.253	0.306	72	>200
+ IP Acid Crosses	0.121	0.371	0.673	84	106
+ IP Acid Movement	0.081	0.300	0.454	84	162
TK10					
Alone Crosses	0.028	0.170	0.142	60	>200
Alone Movement	0.041	0.207	0.195	60	>200
+ IP Acid Crosses	0.014	0.119	0.092	60	>200
+ IP Acid Movement	0.034	0.188	0.166	60	>200
TK33					
Alone Crosses	0.065	0.264	0.305	60	177
Alone Movement	0.038	0.200	0.183	60	>200
+ IP Acid Crosses	0.040	0.204	0.191	60	>200
+ IP Acid Movement	0.041	0.207	0.195	60	>200
TK44					
Alone Crosses	0.135	0.478	0.766	54	58
Alone Movement	0.154	0.427	0.654	54	71
+ IP Acid Crosses	0.105	0.343	0.499	60	107
+ IP Acid Movement	0.186	0.478	0.819	60	58

Supplemental Figure 1. Effects of treatments that yielded a significant Dose x Sex interaction as shown in Supplemental Table S1. Each panel shows data for males and females at each dose. Capital letters M or F indicate that males or females, respectively, had higher scores a the designated dose as indicated by a significant Holm-Sidak post hoc test, p<0.05.

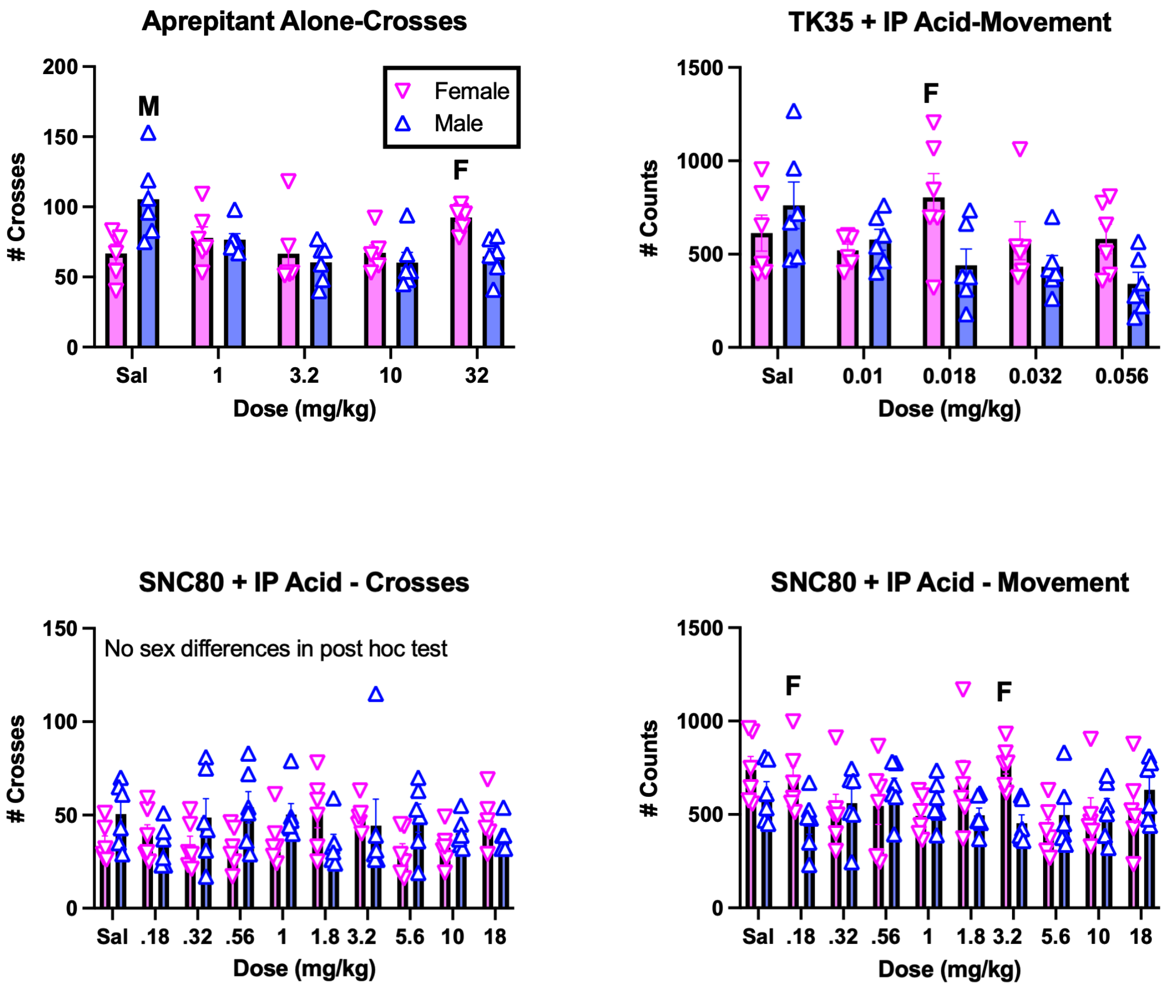

Supplement: Supplementary file 1 [file molecules-29-03331-s001.zip › molecules-3078284-supplementary.pdf]
